# Effects of shell sand burial on seedling emergence, growth and stoichiometry of *Periploca sepium* Bunge

**DOI:** 10.1186/s12870-020-2319-4

**Published:** 2020-03-12

**Authors:** Tian Li, Jingkuan Sun, Hongjun Yang, Jingtao Liu, Jiangbao Xia, Pengshuai Shao

**Affiliations:** Shandong Provincial Key Laboratory of Eco-Environmental Science for Yellow River Delta, Binzhou University, Binzhou, 256600 China

**Keywords:** The Yellow River Delta, Burial depth, Seed germination, Biomass, Nutrient balance

## Abstract

**Background:**

Sand burial plays an irreplaceable and unique role in the growth and distribution of vegetation on the Shell Dike Island in the Yellow River Delta. There are still some unknown on the effects of sand burial on the morphology, biomass, and especially the stoichiometry of *Periploca sepium*, as well as the relationship between these factors.

**Results:**

Shell sand burial depth had a significant influence on seedling emergence, growth, and biomass of *P. sepium*. Shallow sand burial shortened the emergence time and improved the emergence rate, morphological and biomass of *P. sepium* compared to deep burial and the control. Burial depth significantly affected the nitrogen (N) and phosphorus (P) contents of the leaves. With deep burial, the carbon/nitrogen (C/N) and carbon/phosphorus (C/P) ratios decreased firstly and then increased with depth, while the nitrogen/phosphorus ratio (N/P) presented the contrary trend. Correlation analysis showed that the stoichiometry of N/P was positively correlated to morphology and biomass of *P. sepium* at different burial depths. Structural equation model analysis revealed that N was the largest contributor to *P. sepium* biomass.

**Conclusions:**

Optimal burial depth is beneficial to the seedling emergence, growth and nutritional accumulation of *P. sepium*. Stoichiometry has an important influence on the morphological formation and biomass accumulation.

## Background

The Shell Dike Island, which locates in the southwest coast of the Bohai Sea in China, is formed by the confluence of the Yellow River and the Bohai Sea Estuary. It is the only shell dike in the world produced by the coexistence of old and new shells. The dike formed from the shells and debris of shellfish living in the intertidal zone, which has been carried by waves and deposited near the high-tide line. The wildlife resource in the dike and surrounding intertidal wetlands provides rich biological diversity and, is vital to studies of biodiversity and marine ecology [1]. Affected by human disturbance and natural environment, the ecosystem degradation of Shell Dike Island is becoming more and more serious. Therefore, it is the main measure to restore the degraded ecosystem by using vegetation. The vegetation is dominated by shrubs and herbaceous plants, and the variation in sand burial depth caused by sediment buildup in the Yellow River Estuary is the main limiting factor for plant growth [2].

Sowing and afforestation are important methods to restore the vegetation of the sandy land [3]. Compared to other areas, the sandy substrate of Shell Dike Island is easily eroded by water flow. The different burial depths influence the growth conditions of plants, such as temperature, humidity, and other physical factors [4–6]. Seedling emergence is affected by many factors, but optimal sand burial depth is required for germination and excavation [7].

Carbon (C), nitrogen (N), and phosphorus (P) are the three main biological elements in plants [8], and these elements are involved in numerous mutual and inseparable roles in plants [9]. Stoichiometry of these elements affects the nutrient levels, growth, and development of plants, and their integrated effects influence major ecosystem processes [10–12]. Thus, the stoichiometry of plants is of great interest not only for clarifying the distribution and utilization of plant nutrients but also for investigating the nutrient supply and demand within ecosystems [13, 14].

*P. sepium*, a deciduous shrub, is a dominant species with excellent medicinal value and plays an important role in the ecological protection of the Shell Dike Island. Due to human and natural factors, the distribution of *P. sepium* on the Shell Dike Island decreased. Therefore, it is important to study the physiological and ecological factors affecting *P. sepium* to protect and restore the ecology of the Shell Dike Island. Previous studies of the Shell Dike Island mainly focused on plant physiology, soil physical and chemical properties, and the biological diversity [15, 16]. However, few studies have been conducted to investigate the stoichiometry of the dominant plants in this area, and no study has examined how the growth and biomass of *P. sepium* seedling could be influenced by elemental stoichiometry. Therefore, the aim of this study was to evaluate the effects of shell sand burial depth on the seedling emergence, morphology, biomass allocation, and nutrient content of C, N and P in leaves. Our specific objectives were to: (i) quantify the response of seedling emergence, morphology, and biomass allocation to shell sand burial depth; (ii) determine how leaf C, N, P concentrations and the leaf C: N: P ratio respond to different burial depths; (iii) identify the relationships between C, N, P concentration and total biomass. Our results would provide scientific support for the coastal environmental restoration. Further, it will provide a reference for judging how the stoichiometry affect plant biomass.

## Results

### Seedling emergence response to sand burial

**Fig. 1:**
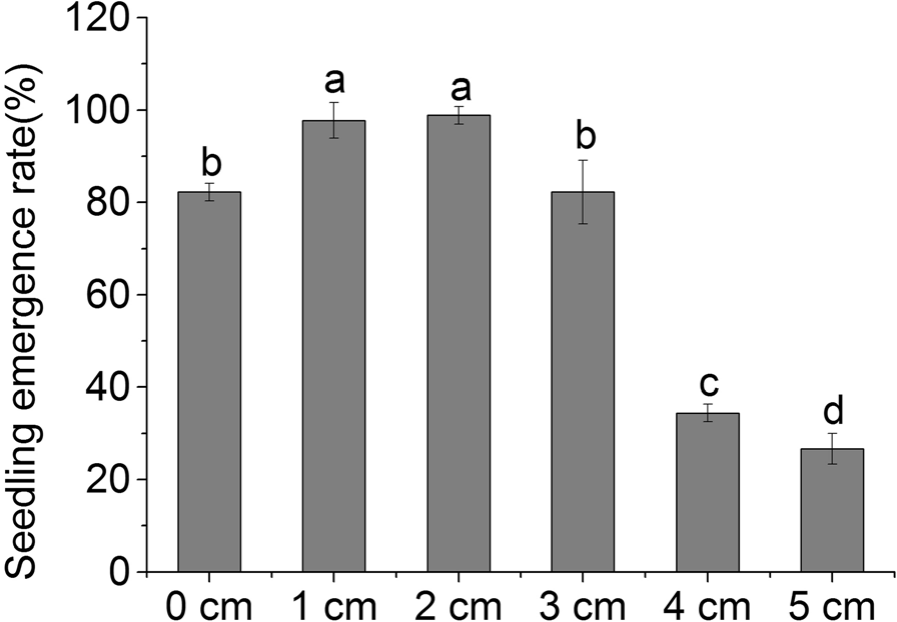
Effects of different burial depths on the percentage of *P. sepium* seedling emergence. Different letters denote significant differences at *P* < 0.05

With increasing shell sand depth, the emergence rate of *P. sepium* seedlings first increased and then decreased (Fig. 1). There was a high rate of emergence under shallow burial conditions (0–3 cm), the highest rate of emergence of 98.9% was observed at 2 cm, and was significantly higher than the untreated control (20.3%, *P* < 0.05). Burial depth of 4–5 cm inhibited seedlings emergence, emergence rate was only 26.7% of the depth of 5 cm, which was significantly lower than the untreated control (67.5%, *P* < 0.05).

**Fig. 2:**
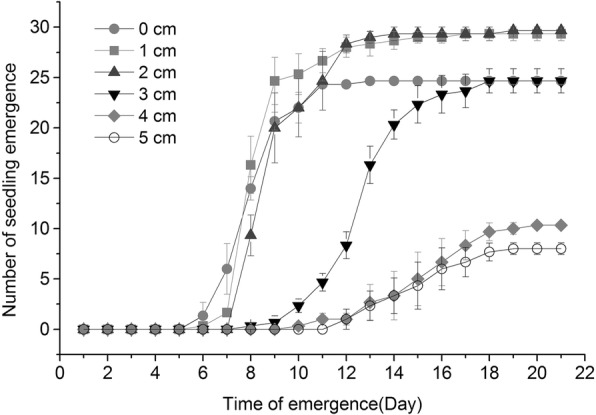
Effects of different burial depths on the process of *P. sepium* seedling emergence

Burial depth had a significant influence on the seedling emergence process. Increased sand burial depth generally led to slower emergence rates (Fig. 2). The emergence speeds at 0–3 cm depth were faster than at 4–5 cm depths. At shallow burial depths, the germination numbers reached the maximum values in the 12th day, but at 4–5 cm depths, the maximum values occurred in the 18th day. The differences of the initial emergence time also indicated *P. sepium* was suitable for shallow burial depth. Burial depth significantly affected the initial emergence time of *P. sepium* seedlings (Fig. S1). The shortest emergence time was 6.7 d when the seeds were buried at 0 or 1 cm. However, there was no significant difference in the initial emergence time of the shallow burial groups (0–3 cm). The longest initial emergence time was observed at the 4–5 cm depths, and was significantly longer than the control and other treatment groups (*P* < 0.05). These results showed that shallow sand burial was beneficial to *P. sepium* for shortening the time of first emergence.

### Morphology response to sand burial

**Fig. 3:**
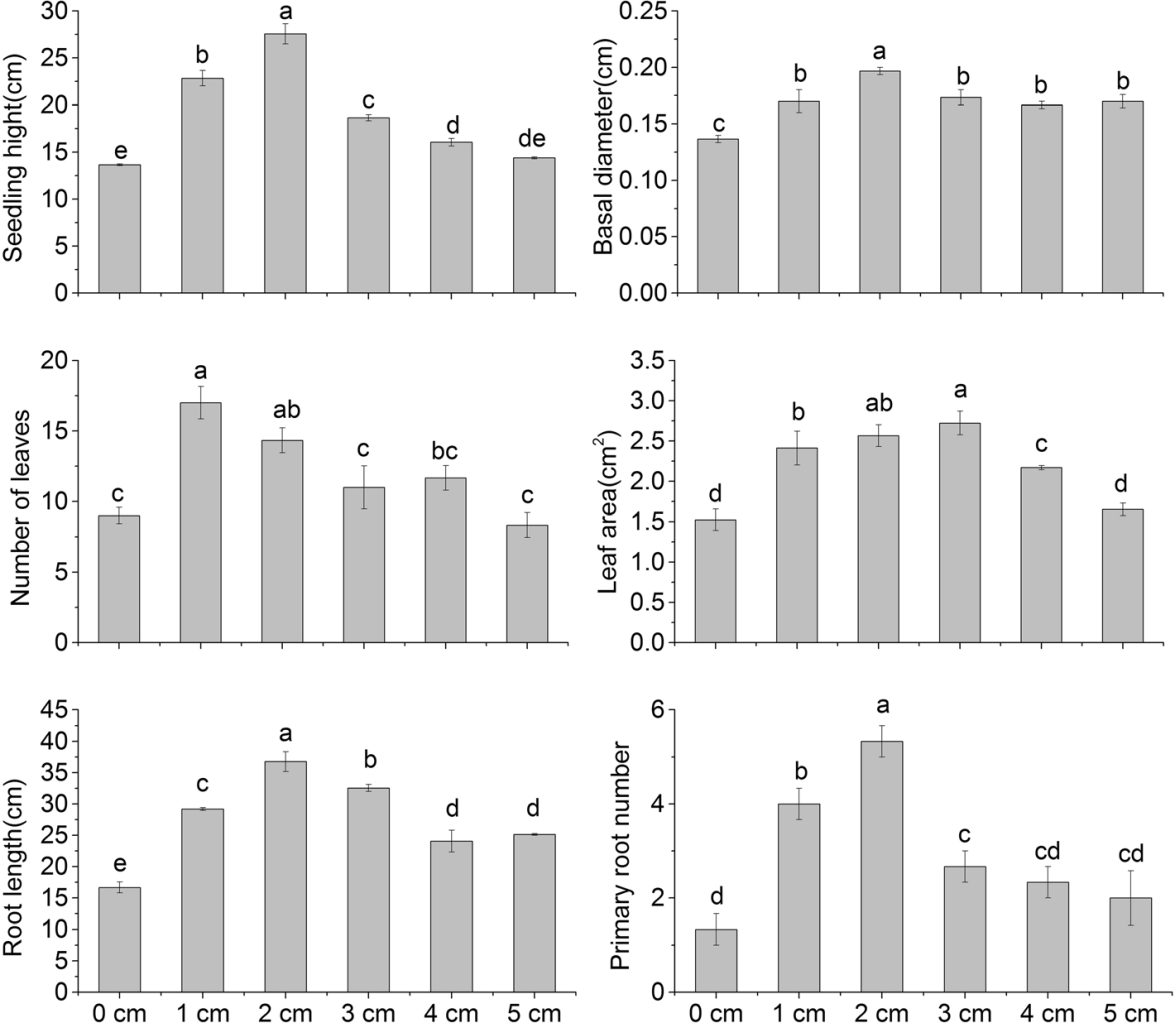
Effects of different burial depths on the height (**a**), basal diameter (**b**), number of leaves (**c**), area of third leaf (**d**), root length (**e**) and primary root number (**f**) of *P. sepium* seedlings. Different letters denote significant differences at *P* < 0.05

Seedling height, base diameter, leaf number, the third leaf area, root length and primary root number were measured for plants buried at different depths. Increasing shell sand burial depth initially led to increasing seedling height, but deeper burial depth resulted in decreasing height (Fig. 3a). The plant height of seedlings at 2 cm depth (27.57 cm) was significantly higher than that of the other treatment groups and the untreated control (13.9 cm). Plant height was the shortest at 5 cm burial depth, but there was no significant difference with the control group. The base diameter of the seedlings in different burial depths showed a decreasing order of 2 cm > 3 cm > 4 cm > 1 cm = 5 cm > 0 cm (Fig. 3b), and all the treatments were significantly larger than the control. The maximum base diameter was 0.197 cm at 2 cm depth (0.06 cm larger than the control group). There was no significant difference between the 1, 3, 4, and 5 cm treatment groups.

The leaf characteristics of *P. sepium* varied with shell sand depth, the number of leaves initially increased with depth but decreased at larger depths (Fig. 3c). Leaf number was greatest at 1 cm depth (17 leaves) and smallest at 5 cm depth. The area of the third leaf followed the same general pattern as the number of leaves, but the differences between treatments were significant (Fig. 3d). The largest leaf area (2.72 cm^2^) was observed at 3 cm burial depth, but this did not differ from the 2 cm treatment group. The leaf area of the control group was 1.52 cm^2^, and did not differ from the 5 cm treatment group.

Burial depth had a significant impact on the growth of the underground part of *P. sepium* (Fig. 3e); the root length increased with the burial depth, showing a trend of 2 cm > 3 cm > 1 cm > 5 cm > 4 cm > 0 cm. The root lengths of all treatments were significantly longer than that of the control group. The longest root length (36.77 cm) was observed in the 2 cm group. The number of primary roots increased with burial depth and the numbers in all the treatment groups were higher than the control group (Fig. 3f). The 0 cm treatment group had the lowest number of primary roots, while the 2 cm group had the most (*n* = 5).

### Biomass and biomass allocation response to sand burial

The effects of different shell sand burial depths on *P. sepium* dry weight were shown in Table 1. The root and leaf dry weight first increased with depth and then decreased. The root dry weight of the 2 cm treatment group was the greatest, and there was no significant difference among the 1 cm and 3 cm, 0 cm and 5 cm groups, respectively. Similar patterns were observed for stem dry weight, and there was no significant difference in 4 cm, 5 cm, and control groups. The values for leaf dry weight decreased in an order of 2 cm > 1 cm > 3 cm > 5 cm > 4 cm > 0 cm, and there was no significant difference among the 1 cm, 3 cm and 5 cm groups, 5 cm, 4 cm and 0 cm groups, respectively.

**Table 1:** Effects of different burial depths on *P. sepium* dry weights of different organs

Depth of shell sand (cm)	0	1	2	3	4	5
Dry weight of root(g)	0.130 ± 0.006^d^	0.287 ± 0.019^b^	0.393 ± 0.015^a^	0.270 ± 0.012^b^	0.213 ± 0.015^c^	0.150 ± 0.006^d^
Dry weight of stem(g)	0.068 ± 0.004^c^	0.113 ± 0.007^b^	0.260 ± 0.006^a^	0.107 ± 0.007^b^	0.090 ± 0.015^c^	0.075 ± 0.003^c^
Dry weight of leaf(g)	0.087 ± 0.003^c^	0.130 ± 0.006^b^	0.193 ± 0.009^a^	0.117 ± 0.017^b^	0.110 ± 0.006^c^	0.113 ± 0.003^bc^

Different letters indicate significant differences among burial depths within organs (*p* < 0.05)

**Fig. 4:**
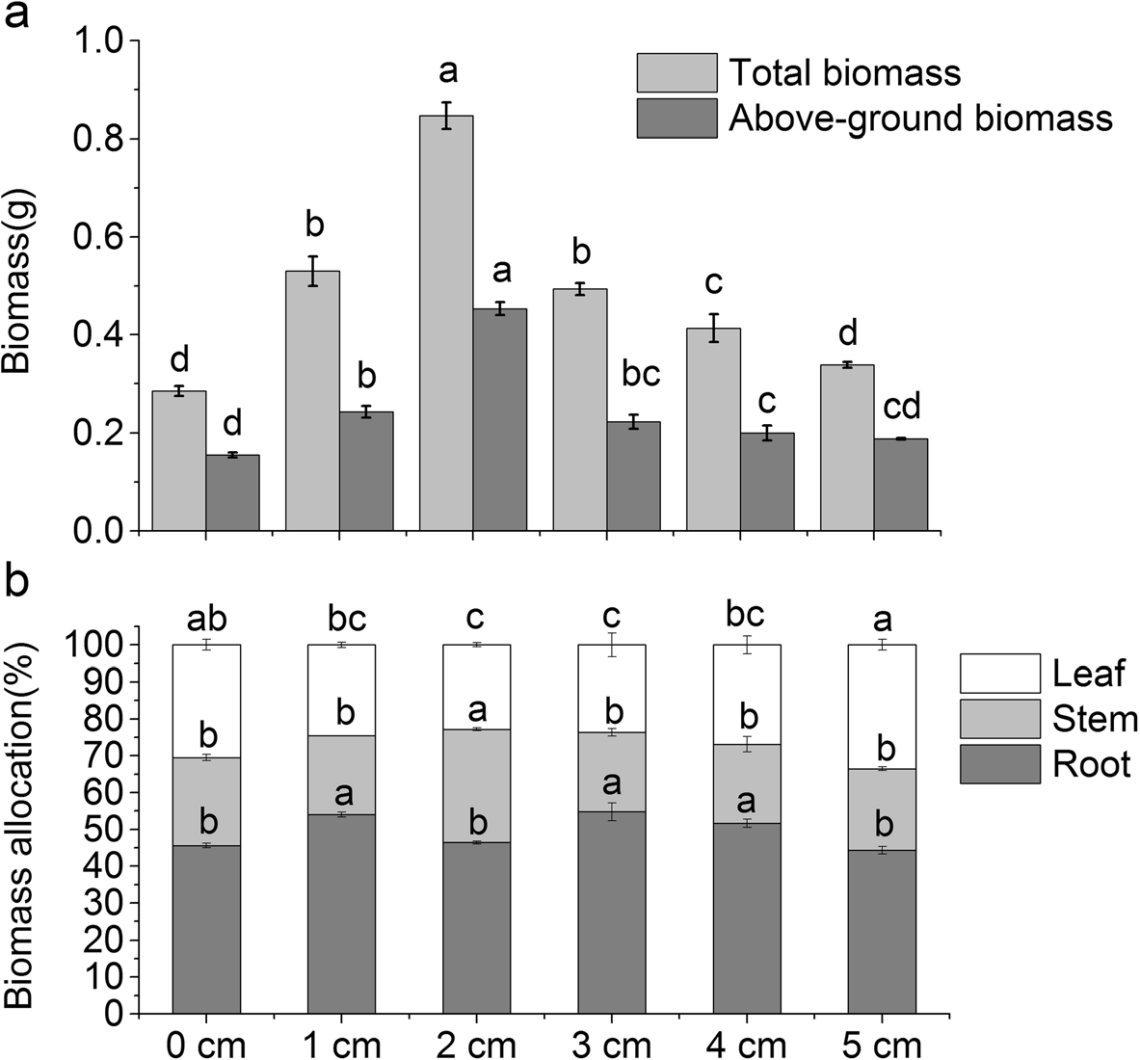
Effects of different burial depths on *P. sepium* seedling biomass (**a**) and distribution (**b**)

The overall and above-ground biomass of *P. sepium* first increased with burial depth and then decreased (Fig. 4a), and different burial depths had significant effects on the biomass structure of *P. sepium* (Fig. 4b). In terms of biomass distribution, the leaf ratios of *P. sepium* decreased at 2 cm and 3 cm, then increased at 5 cm, but had no significant difference with control. The root ratios increased with burial depth from 1 cm to 4 cm. Variation in the stem ratio was not significant except at 2 cm burial depth, where the stem proportion increased significantly (*p* < 0.05).

### Leaf C:N:P stoichiometry response to sand burial

**Fig. 5:**
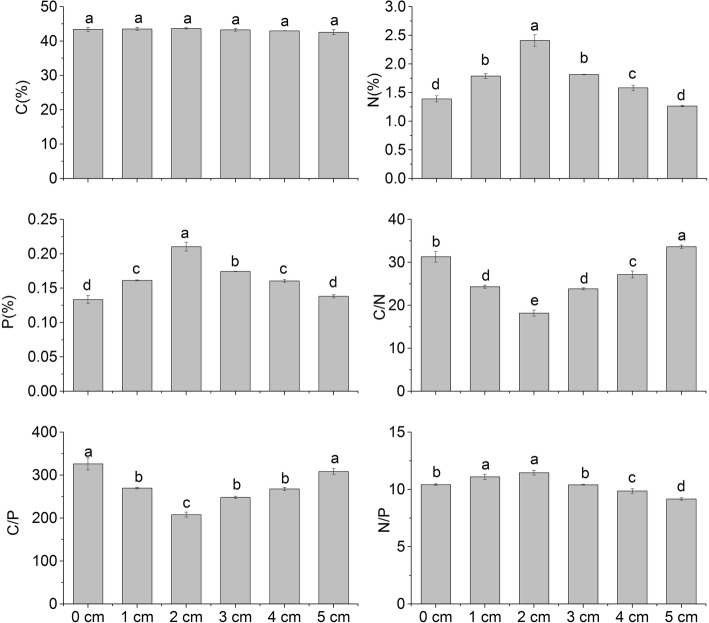
Effects of different burial depths on C, N, and P contents and C/N, C/P and N/P of *P. sepium* leaves

C, N, and P are the main nutrient elements of plants. The C content of *P. sepium* leaves did not vary with burial depth, but the N and P contents first increased and then decreased with burial depth (Fig. 5). The N and P contents of 2 cm treatment group were higher than the other groups, with a value of 2.409% and 0.210%, respectively. There was no significant difference in the N contents of plants buried at 1 cm and 3 cm. At 5 cm, the N content was low (1.265%), but was not significantly different from the control group. The P content did not differ at 1 cm and 4 cm, but was still slightly higher than the control group, and had no significant difference with the 5 cm group.

The stoichiometry of C, N, and P revealed that the C/N and C/*P* values in the treatment groups first decreased and then increased with burial depth (Fig. 5). C/N varied among the treatment groups, there was no significant difference between 1 cm and 3 cm, but C/N at 5 cm was higher than the control group. The minimum C/N and C/P occurred in the 2 cm group, with a value of 18.18 and 208.06, respectively. The maximum C/N was 33.63 at 5 cm depth, which was higher than the control group (2.34). The maximum value of C/P was in the control group (326.03). The N/P differed among the treatment groups, first increased and then decreased with burial depth (Fig. 5). The smallest N/P was observed at 5 cm, followed by the 4 cm group, and the highest N/P was observed at 1 cm and 2 cm depth, but the differences were not significant.

### Relationships between leaf nutrient traits and biomass

There were significant correlations among the stoichiometry, seedling morphology, and biomass at different shell sand burial depths (Table 2). The N content of the leaves was positively correlated with the P content, and the contents of N and P were significantly positively correlated with seedling morphology and biomass index. The increases in leaf N and P contents were related to an increase in total and above-ground biomass, and promoted seedling morphogenesis. C/N and C/P were negatively correlated with the above mentioned morphological and biomass characters while N/P was positively correlated with all of the measurements except base diameter. The results indicated that the stoichiometry of the *P. sepium* leaves influenced the morphogenesis and biomass accumulation of *P. sepium* seedlings at different burial depths.

**Table 2:** The correlation between morphological indexes, biomass and stoichiometry of *P. sepium* leaves with different burial depths

	N	C	P	C/N	C/P	N/P	DW (leaf)	Number of leaves	Area of leaf	DW (stem)	DW (root)	Primary root number	Height	Basal diameter	Total biomass	Above-ground biomass
N	1															
C	0.419	1														
P	.970^**^	0.328	1													
C/N	−.972^**^	−0.347	−.949^**^	1												
C/P	−.919^**^	−0.193	−.975^**^	.942^**^	1											
N/P	.823^**^	.535^*^	.665^**^	−.815^**^	−.584^*^	1										
DW (leaf)	.796^**^	0.225	.805^**^	−.728^**^	−.757^**^	.544^*^	1									
Number of leaves	.600^**^	0.457	.534^*^	−.630^**^	−.522^*^	.635^**^	.590^**^	1								
Area of third leaf	.763^**^	0.252	.789^**^	−.840^**^	−.835^**^	.573^*^	.579^*^	.622^**^	1							
DW (stem)	.905^**^	0.298	.893^**^	−.827^**^	−.819^**^	.677^**^	.898^**^	.482^*^	.545^*^	1						
DW (root)	.921^**^	0.305	.915^**^	−.928^**^	−.905^**^	.723^**^	.834^**^	.671^**^	.788^**^	.899^**^	1					
Primary root number	.843^**^	0.24	.812^**^	−.816^**^	−.794^**^	.688^**^	.851^**^	.685^**^	.681^**^	.810^**^	.850^**^	1				
Height	.916^**^	0.337	.859^**^	−.898^**^	−.830^**^	.814^**^	.844^**^	.707^**^	.718^**^	.890^**^	.945^**^	.918^**^	1			
Basal diameter	.675^**^	0.121	.754^**^	−.629^**^	−.751^**^	0.301	.794^**^	.514^*^	.619^**^	.725^**^	.776^**^	.697^**^	.691^**^	1		
Total biomass	.927^**^	0.299	.922^**^	−.890^**^	−.882^**^	.701^**^	.922^**^	.614^**^	.693^**^	.970^**^	.972^**^	.869^**^	.943^**^	.792^**^	1	
Above-ground biomass	.888^**^	0.279	.883^**^	−.812^**^	−.817^**^	.646^**^	.956^**^	.532^*^	.571^*^	.988^**^	.898^**^	.844^**^	.895^**^	.767^**^	.976^**^	1

** Significant correlation at 0.01 levels (bilateral). * Significant correlation at 0.05 levels (bilateral). DW-Dry weight

The correlation coefficients of the measurements showed that the N content of the leaves had a stronger effect on the leaf number, stem and root dry weights, primary root number and plant height than P. Correspondingly, P was a stronger indicator of seedling morphological characters such as the third leaf area, leaf dry weight and base diameter. However, N and P had similar correlation coefficients for total and above-ground biomass. The close relationships between stoichiometry, seedling morphology and biomass suggested that there were differences in nutrient utilization at different burial depths, which may be a positive response to sand stress. The stoichiometry of the leaf may be the major factor influencing *P. sepium* growth and development at different depths.

**Fig. 6:**
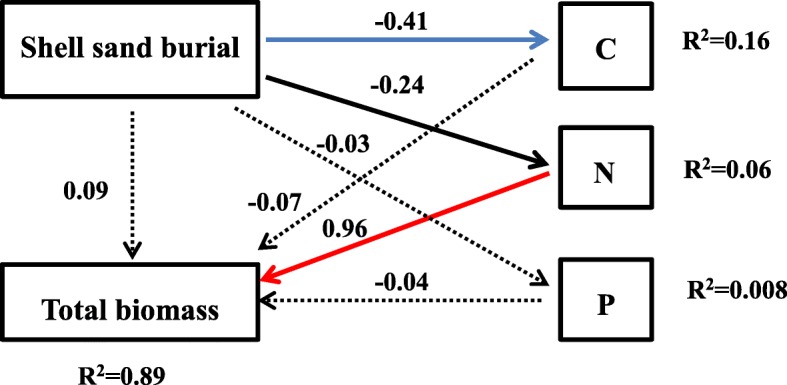
Path diagrams showing the relationships between burial depth, total biomass and C, N, and P contents of *P. sepium* leaves (Black Solid paths are statistically different at *P* < 0.05, blue Solid paths are statistically different at *P* < 0.01, red paths are statistically different at *P* < 0.001, dashed lines indicate non-significant at *P* > 0.05)

The relationships between the C, N, and P contents, total biomass and burial depth were further analyzed by structural equation model (Fig. 6). It is remarkable that burial depth can indirectly affect total biomass by influencing the contents of C, N, and P in the leaves. Burial depth had no significant effect on the P content, but significantly affected C and N. However, there was no significant effect of C on plant biomass; only the relationship between N content and biomass was significant (*P* < 0.001), with a standardized path coefficient of 0.96. This result showed that the N element in the leaf was the major contributor to the biomass of *P. sepium*, while C and P did not play a significant role.

## Discussion

Sand burial influences the growth and distribution of vegetation on the Shell Dike Island in the Yellow River Delta, and plants have a certain tolerance limit to sand burial. If this factor does not exceed the tolerable limit of the plant, it will promote plant growth and development. However, if the burial depth exceeds tolerable limit, it will inhibit the normal growth and development of the plant [2]. Seed germination and emergence can be affected by different burial depths [17, 18]. Seed germination and seedling emergence are vulnerable and sensitive stages in the life history of *P. sepium*, both of which are susceptible to external factors. Therefore, the experiment on the response of the seeds and seedlings to the depth of shell sand can reflect the survival, formation and distribution of *P. sepium* under shell sand burial.

The relationship between the burial depth and the rate of seed germination and seedling emergence is important for quantifying the effect of shell silt accumulation [19]. Our results are consistent with the conclusions of previous studies, where the seed germination and seedling emergence rates were higher at shallow burial depths, and the germination rate decreased with the increasing of sand burial depth, and the seedling emergence delay [20]. Seedling emergence and growth not only depend on the characteristics of the plant species, such as seed weight and size [21], but also the growing environment (i.e., light, water, temperature, soil permeability, etc.) [22–24]. The low rates of seed germination and seedling growth at deep depths observed in this study are considered to be related to similar factors. For example, the small seeds of *P. sepium* have small material energy. When buried too deep, it is impossible for the seeds to unearth properly due to energy material depletion. Previous study has shown that excessive burial depth reduced the permeability of shell sand, led to seed dormancy, and affected the formation of seedlings [25]. Deep burial decreased light intensity and temperature, both of which are unfavorable for seed germination and seedling emergence, although, seedling emergence does not vary between light and dark conditions for some plant seeds [26]. Light conditions have obvious differences in the morphological construction of unearthed seedlings [22, 26]. Water plays a more important role in seed germination than light, as the water storage capacity of shell sand is poor, and evaporation leads to low water content at the surface. The water content of shell sand increases with increasing burial depth. Adequate moisture is beneficial to seed germination and seedling emergence, but too high moisture, especially when the permeability is poor, may inhibit seed respiration, and inhibit seedling emergence. It could also result in seed decay. The correlation analysis revealed a positive correlation between the morphology index and the NP stoichiometry of the *P. sepium* leaves under sand burial, indicating that the stoichiometry of the leaves also played an important role in seedling morphology. This role may be very significant due to the fact that variation in the burial depths changed the microenvironment at the time of seedling emergence, and that seeds are sensitive and critical to changes in environmental conditions, and thus, affecting the morphogenesis of seedlings.

Sand thickness has an important influence on the biomass distribution of plant seedlings [27]. The results presented here showed that the proportion of leaf biomass reduced and then increased with increasing of shell sand depth, while the proportion of underground biomass increased and then decreased. Interestingly, the seedlings had the largest total biomass at 2 cm depth, but their underground ratio was similar to plants grown at 5 cm and 0 cm, which had the lowest total biomass. It is due to the fact that the above-ground biomass of *P. sepium* is distributed more to the stem when buried at 2 cm, resulting in an increase in above-ground biomass and a decrease in the proportion of below-ground biomass. This is consistent with studies which showed that sand burial changed the biomass distribution of seedlings and induced seedlings to allocate more resources to the above-ground or below-ground parts. For example, sand burial can reduce the biomass ratio of *Nitraria sphaerocarpa* on the ground and increase the biomass ratio of underground roots [28]. In *Scaevola plumieri*, sand burial improves stem growth and the replacement of leaf area [29]. However, Shi et al. found that different sand burial depths did not significantly affect the distribution of above and below ground biomass in eucalyptus seedlings [30]. These studies suggest that there are differences in the biomass distribution mechanisms caused by burial depth, reflecting different ecological strategies of plant adaptation. This is attributed to the characteristics of the species and the specific environmental conditions [29].

During plant growth and development, the accumulation of biomass is inseparable from the contents of C, N, and P [31]. In this study, variation in the N content of the leaves was consistent with the biomass change in different burial depths, the N content first increased and then decreased with depth. This relationship may enhance the photosynthetic characteristics of plants and promote the accumulation of products [31]. At the same time, the stoichiometry of a plant may reveal its adaptability to the environment. Generally, a high leaf C content suggests that the plant’s defense ability is strong, while the N and P contents reflect the difference in the plant’s ability to compete with nutrients [32]. Different sand burial depth resulted in significant variations in the contents of N and P and the ratios of C/N, C/P and N/P in *P. sepium* in the present study. These results showed that burial depth had a significant effect on the nutrient utilization of *P. sepium* seedlings. Although the C content in *P. sepium* leaves was the largest at 2 cm depth, the difference between the treatment groups was not significant. The contents of N and P, however, first increased and then decreased with depth. At 2 cm, the contents were the largest and the differences were significant. These results indicated that *P. sepium* regulated the N and P inputs of the leaf tissue, but not the content of C to ensure its normal life activities under the sand burial.

Studies have shown that N and P in plants promote photosynthesis and growth and that the contents of N and P in the leaves are closely related to the chlorophyll content [33]. N or P deficits can inhibit the activity of the optical system in chloroplast cells, resulting in light oxidation stress and photo-suppression reaction [34]. There were strong, positive correlations between biomass and N and P contents of the *P. sepium* leaves. The changes of biomass and N and P content were similar with depth. The above indexes reached their maximum values at 2 cm depth, indicating that this is the optimal depth for biomass accumulation and nutrient utilize. Other studies have shown that stoichiometry affected the distribution of plant biomass, high N content can increase leaf photosynthesis, increase the accumulation of assimilation products, and significantly reduce the root stem ratio (R/S; i.e., increase the proportion of biomass allocated to the upper part of the ground). Conversely, low N content increases the plant R/S [35]. In this study, the stoichiometry of *P. sepium* leaves was unrelated to R/S. Thus, the stoichiometry of *P. sepium* leaves plays an important role in the accumulation of biomass during the seedling stage, but does not play a decisive role in the distribution of biomass during growth. This may be due to the fact that sand burial changes the soil microenvironment around plant roots, such as nutrient, which has an important impact on the distribution of plant biomass [36]. However, the patterns of biomass distribution during plant growth are also influenced by species and underlying genetic factor [37, 38].

The C/N and C/P of plant represent the ability of the plant to assimilate and accumulate C, which reflects the nutrient use efficiency of the plant body. The use of nutrient elements by plants can be reflected by its C/N and C/*P* values [39]. The C/N (22.5) and C/P (232) of the *P. sepium* leaves were higher than the global average levels except the 2 cm burial group in the present study [40, 41]. This shows that the utilization of plant nutrient is high at all burial depths, suggesting a level of tolerance to sand burial. Slightly lower C/N and C/P at 2 cm depth may be contributed by the large biomass, prosperous growth, and great demand for nutrients in this treatment group. However, the limited nutrients of the shell sand matrix may cause nutrition deficits, which affect nutrient absorption. The N/P in the leaf can be used to analyze the relationship between plant growth and nutrient restriction. At all depths, the N/P of *P. sepium* leaves was less than 14. According to the theory of nutritional restrictions [42], the growth and biomass accumulation of *P. sepium* seedlings are mainly limited by N. Judging from the N/P of *P. sepium* at different burial depths, the N/P was the highest at the 1-2 cm depths. These results demonstrate that shell sand burial at1–2 cm is the most favorable for *P. sepium* nutrient absorption*.*

The influences of different shell sand depths were investigated for seedling emergence, growth and nutrient utilization in the present study. However, we did not examine the dynamics of burial depth in different periods of the *P. sepium* life cycle. Future work should investigate how depth affects other stages of growth, development, and adaptation of *P. sepium*.

## Conclusions

There is a direct relationship between the depth of burial and the germination of seeds and seedling emergence. The proper burial depth can provide suitable environmental conditions for the seedling emergence. The seeds may fail to emerge as lack of oxygen or excessive mechanical resistance when the burial depth is too great. The depth of burial not only affects seedling emergence, growth and morphology, but also the seedling’s absorption of nutrient elements. The results of this study demonstrated that (1) shallow burial (1–2 cm) was conducive to seedling emergence, as emergence rate decreased with increasing depth. (2) The morphological characters and biomass of *P. sepium* showed similar patterns of variation with increasing shell sand burial depth. The leaf ratio of *P. sepium* first decreased and then increased with depth, while the root ratio showed the opposite pattern, and the stem ratio did not vary except at 2 cm depth. (3) The C content of the leaves did not change with increasing depth, but the N and P contents initially increased and then decreased. The contents of N and P in the leaves increased first and then decreased. The C/N and C/P first decreased and then increased with depth. The opposite pattern was observed for N/P. (4) There was a strong correlation between the stoichiometry of the *P. sepium* leaves and the seedling morphogenesis and biomass, indicating that the stoichiometry was an important factor in *P. sepium* seedlings morphogenesis and biomass accumulation. The study of stoichiometry and growth adaptation of the dominant plants on the Shell Dike Island under sand burial is of great importance to coastal environmental restoration efforts.

## Methods

### Experimental materials

*P. sepium* seeds and shell sand were collected from May to October 2016 at the Shell Island and the National Nature Reserve in Binzhou (N 38° 13 ′40.4′′, E 117° 56′ 43.7′′). The seeds were collected in the experimental area of the Shell Dike Island. *P. sepium* is a deciduous shrub of the *Asclepiadaceae* family according to the Flora of China, which was formally identified by Professor Jingkuan Sun in our laboratory. The experimental techniques and plant materials used were strictly adhere to regulations of the People’s Republic of China on nature reserves. Ripe *P. sepium* seeds were naturally dried and stored at 4 °C. A voucher specimen has been deposited at the Shandong Provincial Key Laboratory of Eco-Environmental Science for Yellow River Delta, Binzhou University (voucher No. ps^− 20,161,015^-001). The shell sand was sifted to remove impurities.

### Experimental methods

Thirty *P. sepium* seeds were dibbled in a plastic basin filled with shell sand for each pot. Each treatment was repeated 3 times. The depths of seed burial were 0, 1, 2, 3, 4, and 5 cm, according to the methods described by Mou et al. [43]. The seeds were cultivated in greenhouses with day and night temperature of 25 ± 1 °C and 15 ± 1 °C respectively. The relative humidity was maintained at 50 ± 5%. The numbers of emerging seedlings were counted every day after sowing. Ten seedlings were set in each pot 30 days after sowing. The seedlings were watered once every 2 days. The plant height and number of leaves were measured 60 days after sowing. When harvesting, three plants were selected to determine plant height, leaf number, root length, dry weight, primary root number, basal stem and the third leaf area (determined using the methods of Plaut et al. and Parida et al. [44, 45]).

After characterization of the physical parameters, the roots, stems and leaves were packed into self-sealing bags. Then, these materials were washed with distilled water and dried to constant weight. The dry weight of each part was measured and the leaves of *P. sepium* were fully ground to assess the C and N contents using a Vario EL III Element Analyzer (Elementar, Germany). The P content of the leaves was determined by molybdenum-antimony anti-spectrophotometry (perchloric acid - concentrated sulfuric acid) [46].

### Data analysis

Statistical analyses were conducted using SPSS 19.0 software (SPSS Inc., USA). One-way ANOVA and Duncan multiple contrasts were conducted for significance analysis. Pearson correlation analysis was performed on the leaf stoichiometry and the morphological and biomass measurements. Structure equation model was used to analyze the relationship between depth, total biomass, and the leaf contents of C, N, and P by AMOS 17.0.2 software (Amos Development, Crawfordville, FL, USA). The structure equation model was tested using the maximum likelihood (ML) method and the root-mean—square-error of approximation (RMSEA) indicator was used to evaluate the model fit. Origin 9.0 software (Origin Lab Corporation, USA) was used to draw the figures.

## Supplementary information

**Additional file 1: Fig. S1.** Effects of different burial depths on the first emergence time of *P. sepium*. Different letters denote significant differences at *P* < 0.05.

## Data Availability

The datasets used and/or analysed during the current study are available from the corresponding author on reasonable request.

## Abbreviations

C: carbon
C/N: carbon—nitrogen ratio
C/P: carbon-phosphorus ratio
DW: dry weight
ML: maximum likelihood
N: nitrogen
N/P: nitrogen-phosphorus ratio
P: phosphorus
R/S: root stem ratio
RMSEA: root-mean—square-error-of- approximation
