# Supplementary figures and images for: Effects of shell sand burial on seedling emergence, growth and stoichiometry of Periploca sepium Bunge
Source: BMC Plant Biol. 2020 Mar 12;20:112. doi: 10.1186/s12870-020-2319-4 (PMC7069190; doi:10.1186/s12870-020-2319-4)

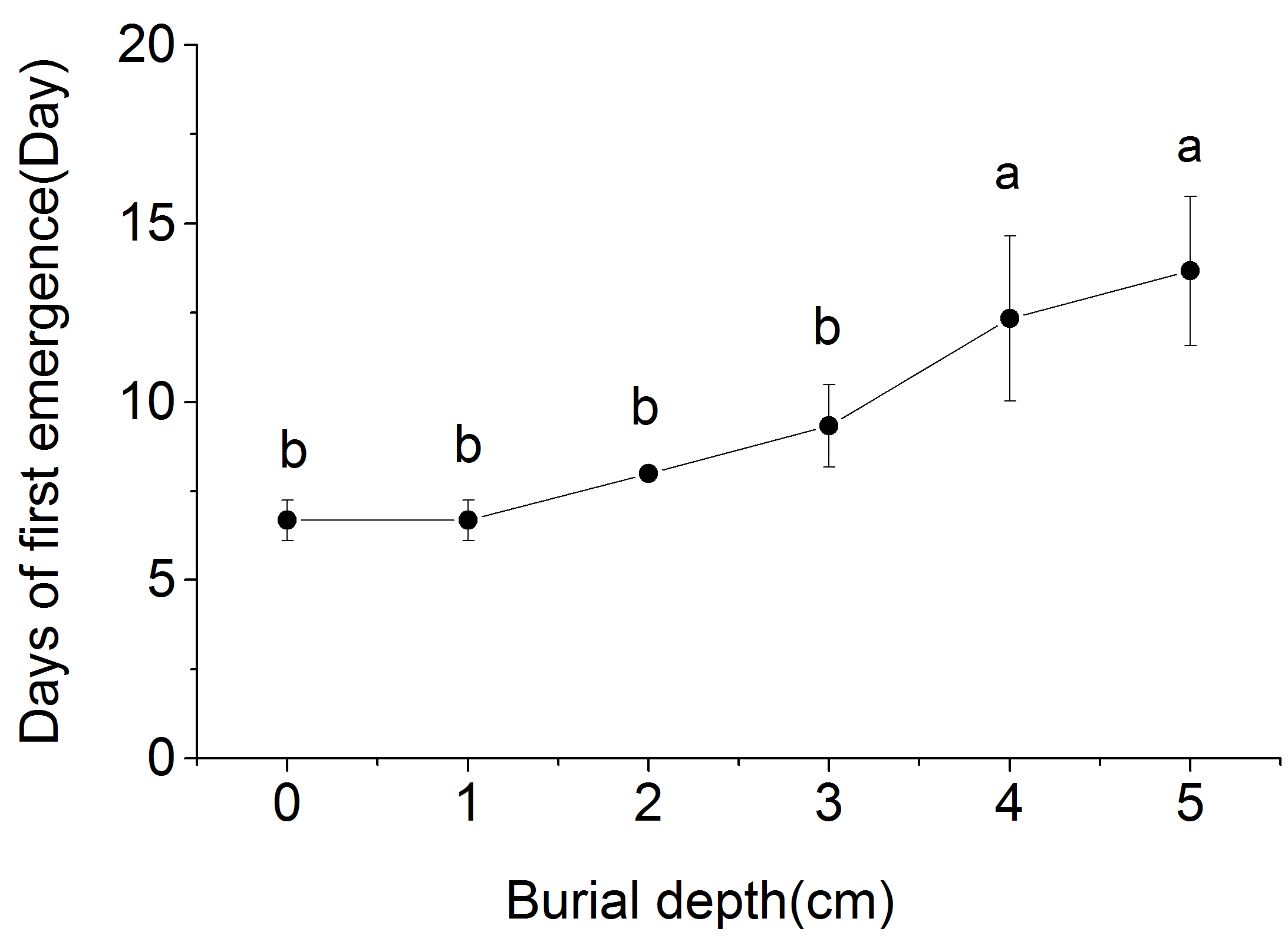

Supplement: Supplementary file 1 — Additional file 1: Fig. S1. Effects of different burial depths on the first emergence time of P. sepium. Different letters denote significant differences at P < 0.05. [file 12870_2020_2319_MOESM1_ESM.jpg]
